# Canal Transportation and Centring Ratio of Paediatric vs Regular Files in Primary Teeth

**DOI:** 10.1016/j.identj.2022.09.003

**Published:** 2022-10-11

**Authors:** Heba Abdelkafy, Alaa M. Eldehna, Nada A. Salem

**Affiliations:** a Endodontic Department, Faculty of Dental Medicine for Girls, Al-Azhar University, Cairo, Egypt; b Pedodontics and Public Health Department, Faculty of Dental Medicine for Girls, Al-Azhar University, Cairo, Egypt; c Department of Pediatric Dentistry and Dental Public Health, Faculty of Dentistry, October 6 University, Cairo, Egypt

**Keywords:** Deciduous root canal, Canal transportation, Centring ratio, Fanta AF baby file, Kidzo Elephant files, ProTaper Next

## Abstract

**Background:**

During mechanical preparation of the primary root canal, the original anatomy of the tooth should be preserved and the instrument should be perfectly balanced centrally into the canal space for reducing the probability of canal transportation. The aim of this research was to compare canal transportation and canal centring ability in primary root canals using ProTaper Next (Dentsply Mailfair,), AF baby (Fanta), and Kidzo Elephant (Endostar, Poldent Sp.) files.

**Materials and methods:**

Eighteen root canals were randomly divided into 3 experimental groups (n = 6 in each group). Instrumentation was performed using ProTaper Next, Fanta AF baby, and Kidzo Elephant files in groups 1, 2, and 3, respectively. During the instrumentation procedure, the irrigation of 2 mL of 1.5% sodium hypochlorite between each file was done, followed by 5 mL of 17% ethylenediaminetetraacetic acid as a final irrigating solution. Cone-beam computed tomography images were obtained before and after instrumentation. Each group was evaluated for transportation and centring ratios.

**Results:**

On comparing all the tested groups within each root canal level canal transportation, the results revealed a statistically nonsignificant difference in the buccolingual direction (P > 0.05). Meanwhile, in the mesiodistal direction, group 1 showed a statistically highly significant difference compared to groups 2 and 3 at the cervical level (*P <* .01). However, in both middle and apical root canal levels, there was a statistically nonsignificant difference amongst all groups (P > 0.05). Regarding the centring ability comparison of the 3 groups within each root canal level, there was a statistically nonsignificant difference amongst all groups (P > 0.05) in both buccolingual and mesiodistal directions.

**Conclusions:**

The ProTaper Next regular rotary file and the paediatric rotary files showed no difference in canal transportation and centring ability in the buccolingual direction, while in the mesiodistal direction at the cervical root canal level, the ProTaper Next showed high transportation liability.

## Introduction

Root canal treatment is indicated for primary teeth displaying signs of irreversible pulpitis or pulp necrosis. Biological and mechanical preparation of the root canal is an important step in the removal of microbes and debris from the root canal in primary teeth, like in permanent ones, to provide a pathway for irrigating solutions to reach the apical third of the root canal, which is referred to as chemomechanical preparation, and to achieve a high level of endodontic treatment success.^1^

In traditional paediatric endodontics, root canal preparation is performed with hand instruments; however, this manual technique may require long chair time for children. Nickel-titanium (NiTi) files were designed for permanent teeth and were initiated by Barr et al to be used in primary dentition.^2^^,^^3^ On comparing the NiTi with the hand files for root canal preparation in primary teeth, the former was found to be faster and has resulted in uniform and predictable fillings.^4^

Recently, paediatric endodontic NiTi rotary file systems have been developed to overcome the difficulties encountered with regular rotary files. Their use allows for safer, convenient root canal preparation; moreover, it minimises the fatigue of the patient and the dental team. On the other hand, the formation of cracks in the root dentin, occurrence of perforations, ledge formation, transportation of canals, and improper taper of the canal preparation are various procedural errors that may arise during the biomechanical preparation of the root canals.^4^^,^^5^

The preservation of the original anatomy and the maintenance of the maximum dentin thickness should be maintained during biomechanical preparation, as the instrument should be perfectly balanced centrally in the canal space for reducing the probability of canal transportation.^4^ The American Association of Endodontics defines transportation as “the elimination of the canal wall structure in the apical half of the canal on the outside curve because of files’ tendency to revert to their initial linear form during intra-canal instrumentation.”^6^ Meanwhile, the centring ability is defined as the ability of the instrument to remain centred in the root canal system during intracanal instrumentation.^7^ So, effective enlargement with the maintenance of the original root canal anatomy is considered another critical factor during the root canal procedure.^5^

The superelastic properties of NiTi rotary instruments have considerably simplified endodontic treatment. For this reason, NiTi instruments are suitable for the preparation of curved root canals with minimal stress and bend to follow the canal's anatomic curvature, resulting in less canal transportation.^8^ ProTaper Next (Dentsply Mailfair) is the successor to the ProTaper Universal system, which has been the gold standard in endodontics for many years. Its innovative off-centred rectangular cross-section gives the file a snake-like “swaggering” movement as it glides through the root canal. The M-Wire NiTi material improves file flexibility while still retaining cutting efficiency. The unique swaggering movement and the greater flexibility of the ProTaper Next files make it possible to shape more severely curved narrow canals than what was possible before.^9^^,^^10^ Fanta AF baby files (Fanta) are designed from advanced metallurgy of H-wire tech with train cross-sectioning, which improves flexibility, cutting efficiency, and cyclic fatigue resistance.^11^ Meanwhile, the Kidzo Elephant file (Endostar, Poldent Sp.) is made of heat-treated CM NiTi wire, which increases the file flexibility and cyclic and torsional fatigue resistance compared to the conventional NiTi alloy. It has a convex triangular cross-section assumed to possess greater cutting efficiency and enough clearance space.^12^

Investigators used several methods such as histologic sectioning, radiography, electron microscopy, computed tomography (CT), cone-beam CT (CBCT), micro-CT, and stereomicroscopy to evaluate the shaping abilities of the endodontic instruments. Recently, CBCT has been widely used for the effective assessment of prepared root canals by endodontic instruments.^13^

So, this study aimed to evaluate the canal transportation and centring ability of 2 paediatric (Fanta AF baby and Kidzo Elephant) files and one regular rotary file (ProTaper Next) in deciduous molar root canals using CBCT. The null hypothesis was that there is no difference in canal transportation and centring ability between regular and paediatric NiTi rotary files.

## Methods

The present is a randomised in vitro study that was performed in the endodontics and paediatric departments of the Faculty of Dental Medicine for Girls, Al-Azhar University, Cairo, Egypt. The research question in this study was addressed in terms of the PICO question, which involves 4 elements: problem (P), intervention (I), comparison, (C), and outcome (O), as follows:

**P.** Canal transportation during intraradicular cavity preparation (problem).
**I**. Root canal preparation using regular ProTaper Next files (intervention).
**C**. Root canal preparation using the paediatric Fanta AF baby file and Kidzo Elephant files (comparison).
**O.** Preservation of normal canal curvature (outcome).

The sample size was calculated on MedCalc version 20.019, and according to a previous study conducted by Jain et al,^14^ adjusting the confidence interval to 95%, the power of the test to 90, and the number of pairwise comparisons to 3, the minimum sample size per group was found to be 4 teeth with a total sample size of 12 teeth divided into 3 groups.

### Sample selection and preparation

Eighteen freshly extracted primary molars were used in this study after obtaining ethical approval from the Research Ethics Committee (REC), Faculty of Dental Medicine for Girls, Al-Azhar University, Cairo, Egypt (Code: REC-PD-22-09). All methods were reported according to the Checklist for Reporting In Vitro Studies guidelines and regulations. Informed consent was obtained from patients’ guardians to use their teeth in this study.

The collected teeth were washed under running water. Any remaining soft tissue fragments were removed from their root surfaces, and teeth were stored in a sterile saline solution.

The selection of root canals was done on the bases of certain inclusion and exclusion criteria. The selected teeth should have at least two-thirds of their root length remaining without any signs of resorption, and there should be no evidence of calcification or internal resorption.

The initial removal of caries was done first, then coronal cavity preparation was made using No. 2 round bur (Mani). K-files #15 (Mani) were used to measure the working length of all teeth by passing them through the apex and then subtracting 1 mm from the apex. All specimens were then randomly divided into 3 groups (n = 6 in each group) according to the rotary instrument used. A random sequence was generated using a random sequence generator website (https://www.random.org/sequences) in 3 groups (groups 1, 2, and 3). Allocation concealment was done by one clinician by inserting each root in a separate envelope, followed by shuffling of envelopes and then writing a number from 1 to 18 on each envelope.

**Fig. 1:**
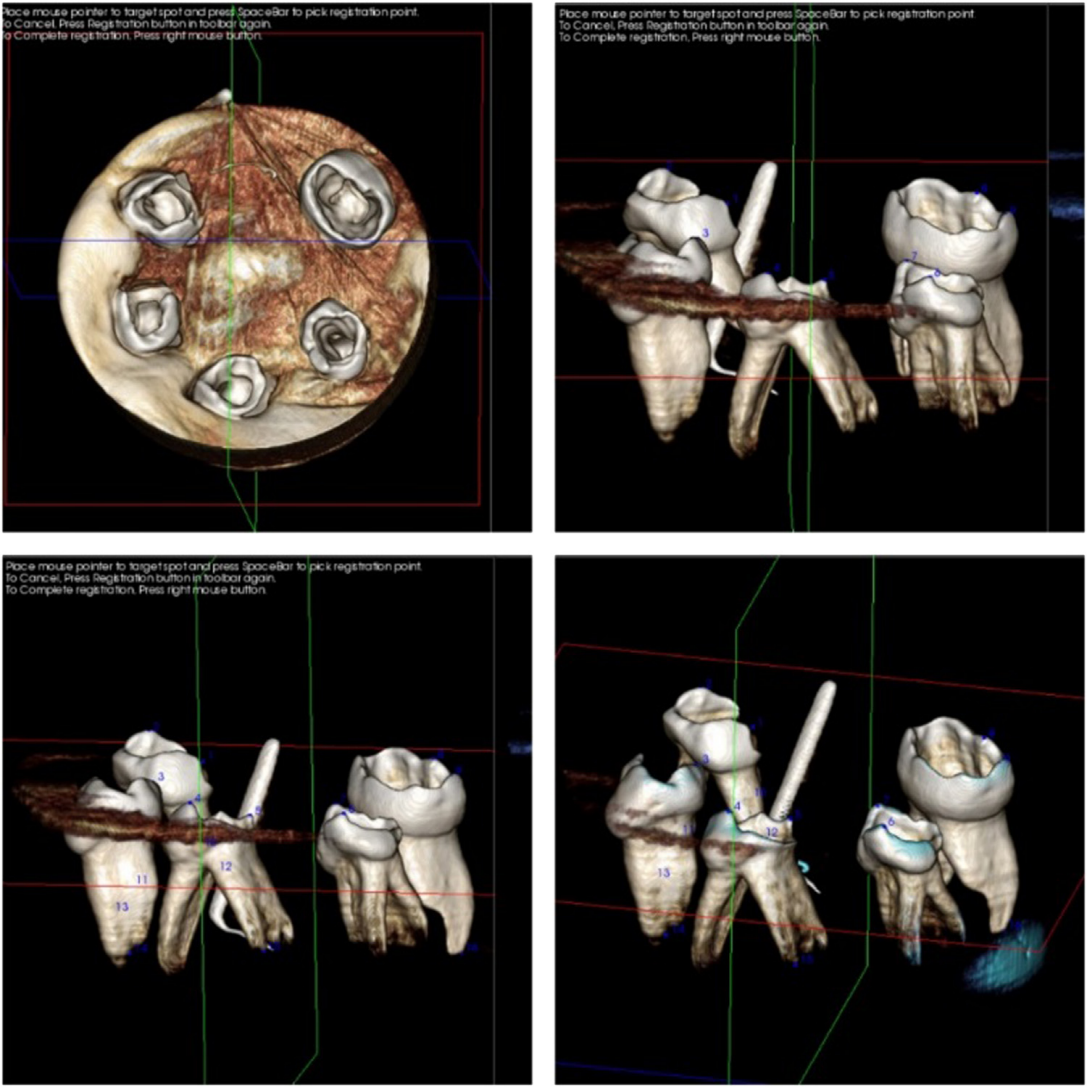
Photograph showing cone-beam computed tomography (CBCT) superimposition that ensures standardisation of pre- and post-CBCT scanning.

To ensure the standardisation of pre- and post-CBCT scanning, each group of teeth was arranged on a rubber base (Zetaflow), which was horseshoe-shaped to accommodate the similarly shaped scanning tray of the CBCT machine (Genoray Papaya 3D). This custom-made rubber base was constructed with dimensions less than the field of view of the CBCT machine. Each group was then submitted to the CBCT examination at 75 kV and 15 mA to determine the canal shape before instrumentation. For each specimen, according to the distance from the cementoenamel junction, 3 tomographs were selected for the coronal, middle, and apical thirds (Figure 1).

Root canal preparation was performed using an endomotor (GeoSoft, EndoEst, Motor-Mini) with an apex locator. During the instrumentation procedure, 2 mL of 1.5% sodium hypochlorite was irrigated between each file, followed by 5 mL of 17% ethylenediaminetetraacetic acid as the final irrigating solution using a conventional syringe and a 30G needle placed 1 mm shorter than the working length. The data used and/or analysed during the current study are available from the corresponding author upon reasonable request.

### Sample grouping

Samples were randomly divided into 3 experimental groups according to the type of rotary file used for mechanical preparation.

- **Group 1 (6 samples):** using ProTaper Next files (X3; #30/0.07).
- **Group 2 (6 samples):** using Fanta AF baby files (#30/0.06).
- **Group 3 (6 samples):** using Kidzo Elephant files (#30/0.06).

After root canal preparation of all samples, they were scanned using CBCT as the pre-instrumentation scanning protocol. The shortest distance between the un-instrumented root's periphery (mesial [M1], distal [D1], buccal [B1], and lingual [L1]) and the instrumented root canal's edge (mesial [M2], distal [D2], buccal [B2], and lingual [L2]) was determined using a length tool on reconstructed cross-sectional images of the pre- and post-instrumentation scanning.

Each group was evaluated for the transportation and centring ratio using the following formulas for each direction^15^:

- Canal transportation (mesiodistal [MD] direction) = (M1 − M2) − (D1 − D2)
- Canal transportation (buccolingual [BL] direction) = (B1 − B2) − (L1 − L2)
- Centralisation ability ratio (MD direction) = (M1 − M2)/(D1 − D2)
- Centralisation ability ratio (BL direction) = (B1 − B2)/(L1 − L2)

### Statistical analysis

Data were collected, revised, coded, and entered into the Statistical Package for Social Science (IBM SPSS) version 23. Quantitative data were presented as mean values, standard deviations, and ranges. Comparisons of quantitative data with nonparametric distributions between more than 2 groups were performed using the one-way analysis of variance. Comparisons of quantitative data with nonparametric distributions between more than 2 paired groups were performed using the Friedman test.

The confidence interval was set to 95%, and the acceptable margin of error was set to 5%. Thus, *P* values >.05 were considered nonsignificant (NS), *P* values <.05 were considered statistically significant (S), and *P* values <.01 were considered highly significant (HS).

## Results

### Canal transportation

**Fig. 2:**
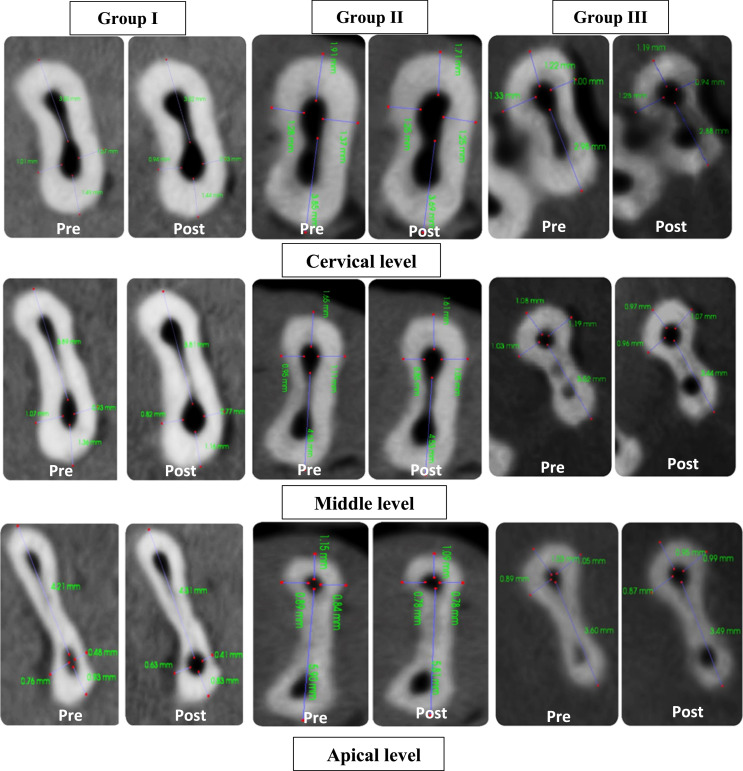
An image displaying preoperative and postoperative axial slices of cone-beam computed tomography showing dentin thickness at mesiodistal and buccolingual aspects of the canal lumen of the 3 groups within each root canal level.

Comparing all the tested groups within each root canal level, results revealed a statistically nonsignificant difference in the BL direction (*P <* .05). Meanwhile, in the MD direction, group 1 (ProTaper Next) demonstrated a statistically HS difference compared to groups 2 and 3 (Fanta AF baby and Kidzo Elephant files) at the cervical level (*P<*.01). However, in both middle and apical root canal levels, there was a statistically nonsignificant difference amongst all groups (*P<*.05; Table 1; Figure 2).

**Table 1:** Mean values and standard deviations for canal transportation buccolingually (BL) and mesiodistally (MD) of all groups within each root canal level.

Canal transportation	Root canal levels	Group 1	Group 2	Group 3	Test value≠	*P* value	Sig.
		n = 6	n = 6	n = 6			
		Mean ± SD	Mean ± SD	Mean ± SD			
**BL direction**	Cervical	−0.03 ± 0.09	0.00 ± 0.04	0.00 ± 0.09	0.391	.822	NS
	Middle	−0.01 ± 0.13	0.04 ± 0.14	−0.04 ± 0.12	1.115	.573	NS
	Apical	0.00 ± 0.11	−0.07 ± 0.21	−0.03 ± 0.05	0.464	.793	NS
	(Friedman) test value	0.087	1.130	0.000			
	*P* value	.957	.568	1.000			
**MD direction**	Cervical	−2.02* ± 1.84	0.11 ± 0.10	0.04 ± 0.09	12.039	.002	HS
	Middle	−0.02 ± 0.12	−0.05 ± 0.05	0.04 ± 0.09	2.281	.320	NS
	Apical	0.02 ± 0.10	−0.03 ± 0.05	0.03 ± 0.10	2.440	.295	NS
	(Friedman) test value	11.565	6.333	0.261			
	*P* value	.003	.042	.878			
	Post hoc analysis						
	Cervical	Group 1 vs group 2	Group 1 vs group 3		Group 2 vs group 3		
		.004	.004		.229		

*P* value >.05: not significant; *P* value <.05: significant; *P* value <.01: highly significant.

≠ : Kruskal–Wallis test, significance (*) post hoc analysis.

### Centring ability

Regarding the comparison of the 3 groups within each root canal level, there was a statistically nonsignificant difference amongst all groups (*P<*.05) in both BL and MD directions (Table 2 and Figure 2).

**Table 2:** Mean values and SDs for centring ability buccolingually (BL) and mesiodistally (MD) of all groups within each root canal level.

Centring ability	Root canal levels	Group 1	Group 2	Group 3	Test value≠	*P* value	Sig.
		n = 6	n = 6	n = 6			
		Mean ± SD	Mean ± SD	Mean ± SD			
**BL direction**	Cervical	0.99 ± 0.86	1.02 ± 0.48	3.40 ± 5.79	0.302	.860	NS
	Middle	1.95 ± 2.19	3.41 ± 4.32	1.10 ± 1.14	0.882	.643	NS
	Apical	0.34 ± 0.75	0.52 ± 1.58	0.74 ± 0.43	1.244	.537	NS
	(Friedman) test value	1.652	1.600	0.000			
	*P* value	.438	.449	1.000			
**MD direction**	Cervical	2.13 ± 1.79	3.27 ± 1.58	1.58 ± 0.86	3.614	.164	NS
	Middle	1.79 ± 2.50	1.18 ± 1.40	1.44 ± 0.72	1.064	.587	NS
	Apical	1.22 ± 0.97	0.79 ± 0.95	2.62 ± 1.36	4.641	.098	NS
	(Friedman) test value	3.600	6.333	4.333			
	*P* value	.165	.042	.115			

*P* value >.05: nonsignificant; *P* value <.05: significant; *P* value <.01: highly significant.

≠ : Kruskal–Wallis test.

## Discussion

Anatomically, deciduous teeth have been considered more complex and challenging than their permanent counterparts, which makes chemomechanical preparation a crucial step.^16^ Because cleaning and shaping efficacy relies on several factors such as irrigating solutions and techniques, instrument type, and instrumentation techniques, various studies have compared the cleaning efficiency of either hand with rotary or even different rotary instruments.^1^^,^^16^^,^^17^

The present study aimed to evaluate the canal transportation and centring ability of different rotary endodontic files, where canal transportation refers to the post-instrumentation deviation in the axis comparable to the original pre-instrumentation axis. On the other hand, the centring ability is the ability of the file axis to be in line with the canal axis, thereby avoiding canal zipping, ledging, or perforation.^18^ In past years, several methods, such as scanning electron microscopy, the serial sectioning technique, optical microscopy, photographic evaluation, and software-based assessments, have been used in measuring canal transportation and the centring ratio. However, these techniques require repositioning of the samples for postscanning, which may result in reading errors.^19^^,^^20^ To overcome these errors, in the present study, CBCT was chosen as the method of evaluation since it is considered one of the best methods used to evaluate such parameters. It provides highly accurate and quantifiable multiplanar images at various cross-sections via a noninvasive method; as such, repeatable and acceptable results are obtained. The amount of canal transportation and the direction involved can be viewed at any level.^15^^,^^19^^,^^21^

Three root levels corresponding to cervical, middle, and apical thirds were chosen to be assessed. Several authors considered the root apex the reference point for evaluating canal centricity.^15^^,^^18^ However, taking into consideration the continuous physiologic root resorption associated with primary roots that consecutively affect root length, cementoenamel junction was considered a reference point for this study in accordance with other studies.^4^^,^^15^

The formula introduced by Gambill et al was used in several previous studies, as they assumed its effectiveness in measuring canal transportation and centring ability.^15^^,^^17^^,^^20^ In the present study, the modified Gambill technique used is different from the original one, as the latter was limited to using 2 measurements along a single plane. Unidirectional measurement may not be able to adequately show the geometric changes in 3 dimensions along the length of the canal. However, the modified Gambill technique enables an 8-point circumferential analysis that allowed for an enhanced assessment of the mechanical action of the file systems.^15^

The null hypothesis tested in the present study was that there is no difference in canal transportation and centring ability between regular and paediatric NiTi rotary files; it was accepted except for the results of canal transportation of group 1 in the MD direction at the cervical level, where ProTaper Next showed the lowest mean value (more canal transportation) compared to groups 2 and 3.

The nonsignificant results of the centring ability in both directions, as well as canal transportation in the BL direction, could be attributed to several similarities amongst the 3 systems. They all worked in a crown-down manner with a rotational motion and an almost similar degree of taper of each system and terminated preparation with the same apical diameter (#30/0.07 for ProTaper Next and #30/0.06 for Fanta AF baby file and Kidzo Elephant file). Moreover, they are all made of heat-treated NiTi alloy, which improves the flexibility and shaping ability of the files, maintaining the original canal shape even in severely curved canals with a lower risk.^17^

On the other hand, the ProTaper Next regular rotary endodontic file produces more canal transportation cervically than the paediatric rotary files in the MD direction, which may be attributed to the rectangular cross-section of the ProTaper Next rotary file compared to a triangular cross-section of both paediatric files. Also, the file length may affect the authority over the file, as the regular ProTaper Next file measures 25 mm while the Fanta AF baby files and the Kidzo Elephant file measure 16 and 18 mm, respectively. Although the file size was standardised to be size 30, the taper of the ProTaper Next rotary file was 0.07 mm while that of the paediatric files was 0.06 mm, which means that the increase in diameter of ProTaper Next rotary file per millimetre will be more than that of both paediatric files at the same length.

## Conclusions

Within the limitations of this study, the ProTaper Next regular rotary file and the paediatric rotary files have the same impact on canal transportation and centring ability buccolingually. However, the ProTaper Next showed higher transportation liability at the cervical level mesiodistally, which may be attributed to the rectangular cross-section and regular length (25 mm) with the 0.07 mm taper of this file compared to the triangular cross-section of both paediatric files and shorter file lengths (16 and 18 mm) with a lesser 0.06 mm taper.

## Recommendations

Paediatric rotary files are highly advisable for the preparation of the primary root canal due to their suitable length and adequate cross-section. Furthermore, other studies may be needed to compare the transportation and centring ability of short-length rotary regular files to that of the paediatric rotary file in deciduous root canals using micro-CT.

## Conflict of interest

None disclosed.

## Appendix Supplementary materials

Image, application 1

Image, application 2

Image, application 3

Image, image 4

## References

[1] Kalita S, Agarwal N, Jabin Z, Anand A. (2021). Comparative evaluation of cleaning capacity and efficiency of Kedo-S pediatric rotary files, rotary ProTaper, and hand K files in primary molar pulpectomy. Int J Clin Pediatr.

[2] Barr ES, Kleier DJ, Barr NV. (2000). Use of nickel-titanium rotary files for root canal preparation in primary teeth. Pediatr Dent.

[3] Pinheiro SL, Araujo G, Bincelli I, Cunha R, Bueno C. (2012). Evaluation of cleaning capacity and instrumentation time of manual, hybrid and rotary instrumentation techniques in primary molars. Int Endod J.

[4] Rathi N, Jain SA, Thosar N, Baliga S, Ahmed F, Mehta J. (2021). Comparative evaluation of cleaning efficiency and apical extrusion of debris using two pediatric rotary endodontic files: an in vitro study. Int J Clin Pediatr Dent.

[5] Seema T, Ahammed H, Parul S, Cheranjeevi J. (2020). Comparative evaluation of dentin removal and taper of root canal preparation of hand K file, ProTaper rotary file, and Kedo s rotary file in primary molars using cone-beam computed tomography. Int J Clin Pediatr Dent.

[6] (2003). American Association of Endodontists. Glossary of endodontic terms. American Association of Endodontists.

[7] de Albuquerque MS, Nascimento AS, Gialain IO (2019). Canal transportation, centering ability, and dentin removal after instrumentation: a micro-CT evaluation. J Contemp Dent Pract.

[8] Tabassum S, Zafar K, Umer F. (2019). Nickel-titanium rotary file systems: what's new?. Eur Endod J.

[9] Patnana AK, Chugh A. (2018). Endodontic management of curved canals with protaper next: A case series. Contemp Clin Dent.

[10] Dentsply Sirona Endodontics. ProTaper Next. Available from:https://assets.dentsplysirona.com/dentsply/web/Endodontics/global-page-templates-assets/download-pdf%27s/protaper-next/PTN%20Brochure%20ROW%20EN.pdf. Accessed October 1, 2022.

[11] Bnaiyan AH, Altinawi MK, Lazkani T, Alzoubi H. (2022). Evaluation time and efficacy of root canal rotary preparation in primary teeth: an in-vitro study. Cureus.

[12] BiBodent Dental Supplies. Available from:www.bibodent.com. Accessed October 1, 2022.

[13] Narayanan LL, Vaishnavi C. (2010). Endodontic microbiology. J Conserv Dent.

[14] Jain S, Rathi N, Thosar N,, Baliga S. (2020). Comparative evaluation of canal transportation and canal centering ability in primary root canals using Pro AF Baby Gold and Kedo-S pediatric endodontic rotary files with cone beam computed tomography- an invitro study. Int J Pure Medical Res.

[15] Gajoum A, Patel E, Munshi IE, Tootla S. (2021). A comparison of root canal transportation and centering ability between WaveOne® Gold and Protaper Next® files, using micro-computed tomography. S Afr Dent J.

[16] Prabhakar AR, Yavagal C, Dixit K, Naik SV. (2016). Reciprocating vs rotary instrumentation in pediatric endodontics: cone beam computed tomographic analysis of deciduous root canals using two single-file systems. Int J Clin Pediatr Dent.

[17] Girgis D, Roshdy N, Sadek H. (2020). Comparative assessment of the shaping and cleaning abilities of M-Pro and Revo-S versus ProTaper Next Rotary Ni-Ti systems: an in vitro study. Adv Dent J.

[18] Karabucak B, Gatan AJ, Hsiao C, Iqbal MK. (2010). A comparison of apical transportation and length control between EndoSequence and Guidance rotary instruments. J Endod.

[19] Maitin N, Arunagiri D, Brave D, Maitin SN, Kaushik S, Roy S. (2013). An ex vivo comparative analysis on shaping ability of four NiTi rotary endodontic instruments using spiral computed tomography. J Conserv Dent.

[20] Gambill JM, Alder M, Carlos E (1996). Comparison of nickel-titanium and stainless steel hand-file instrumentation using computed tomography. J Endod.

[21] Elsherief SM, Zayet MK, Hamouda IM. (2013). Cone-beam computed tomography analysis of curved root canals after mechanical preparation with three nickel-titanium rotary instruments. J Biomed Res.

