# Supplementary material for: Canal Transportation and Centring Ratio of Paediatric vs Regular Files in Primary Teeth
Source: Int Dent J. 2022 Oct 11;73(3):423–9. doi: 10.1016/j.identj.2022.09.003 (PMC10213759; doi:10.1016/j.identj.2022.09.003)
Supplement: Supplementary file 1 [file mmc1.docx]

**Assignment number:** EKBEAW-3802

**Filename:** Paper Total - modified - Clear_INQ-6154814622_EKBEAW-3802.doc

**Primary Editor Name:** Jeffrey K


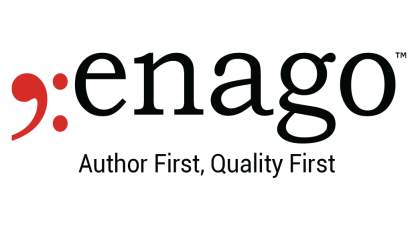


**^[[1]](#footnote-1)^ In case of any clarifications or questions please approach us at** [**submit@enago.com**](mailto:submit@enago.com)

Dear Author:

Thank you for entrusting us with your manuscript and opting for our Copyediting service.

We have edited your assignment EKBEAW-3802 for language and grammar, from the aspect of fluency, and would like to share our experience in editing your manuscript.

Overall, we have made moderate revisions to the manuscript in terms of language and grammar. We have also made some sentence reconstructions and word-choice changes for accuracy and enhanced clarity. We hope that the revisions meet your expectations from our service.

Further, we have checked the formatting of your manuscript thoroughly against the guidelines. Considerable formatting changes were required for adherence to the guidelines. Please address the notes given in the “Journal Formatting” section for complete adherence.

Please be assured that we have edited the manuscript to the best of our ability and have clarified some of our changes through remarks. As a step toward finalization, we suggest that you resolve all remarks in the main file and separated files, as this is important for successful publication.

We have added tips on academic writing conventions specific to your manuscript in the edited file. We hope you find these tips helpful for future writing.

We wish you the very best for the successful publication of your manuscript and look forward to working with you again.

Sincerely,

Your Editor

Jeffrey K

**Journal Formatting**

- The manuscript has been formatted as per journal guidelines. Some sections are moved and rearranged as per the journal’s requirement. Please check.
- The journal encourages authors to provide an ORCID iD when submitting a manuscript.
- Note that the manuscripts should be uploaded as Word (.doc) or Rich Text Format (.rft) files as per the journal. Thus, we have converted your manuscript in the .doc extension format.
- Please suggest the names and current e-mail addresses of three potential international reviewers whom you consider capable of reviewing your manuscript. In addition, please suggest one of the associate editors to review your manuscript.

**Note**

Two-byte fonts such as Mincho and SimSun should not be used in documents as they are not processed by most software, and thus, are not accepted by journals. Please use a suitable font, e.g., Times New Roman or Verdana, based on the journal guidelines.

1. **^1^Editor Message is a means of personal communication between the author and the editor through which the editor clarifies manuscript-related points, provides suggestions/improvements, and/or notifies the author about the next steps.** [↑](#footnote-ref-1)
