# Supplementary material for: Canal Transportation and Centring Ratio of Paediatric vs Regular Files in Primary Teeth
Source: Int Dent J. 2022 Oct 11;73(3):423–9. doi: 10.1016/j.identj.2022.09.003 (PMC10213759; doi:10.1016/j.identj.2022.09.003)

**Editors Comments**

| **Comments** | **Authors Reply** |
| --- | --- |
| The length of the article title should not exceeds 75 characters. Please edit and provide the same in all the stage of manuscript submission. | I changed the title into 85 characters as mentioned in the box of the full title. |


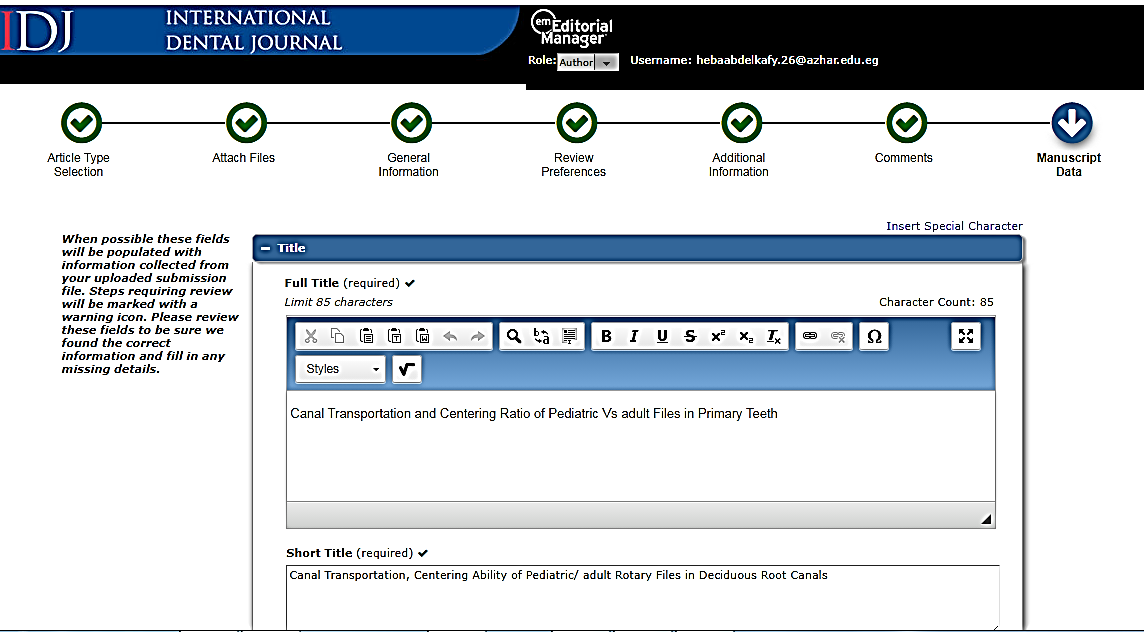

Supplement: Supplementary file 2 [file mmc2.docx]
