# Supplementary material for: Canal Transportation and Centring Ratio of Paediatric vs Regular Files in Primary Teeth
Source: Int Dent J. 2022 Oct 11;73(3):423–9. doi: 10.1016/j.identj.2022.09.003 (PMC10213759; doi:10.1016/j.identj.2022.09.003)
Supplement: Supplementary file 3 [file mmc3.pdf]

Sample size calculation:

Sample size was calculated on MedCalc program version 20.019 and according to a previous study done by ***Jain et al. (2020)*** who comparing in his study the canal centring ability in primary root canals using Pro AF Baby Gold and Kedo-S pediatric endodontic rotary files with Cone Beam Computed Tomography and found that the centring of maxillary molar tooth at the mesial root at level 6 mm from CEJ was found 1.21±0.56 in group I and 2.83±0.75 in group II with statistically significant difference between both groups at p-value <0.001; adjusting the confidence interval to 95%; power of the test to 90; number of pairwise comparison to 3; ***the minimum sample size per group was found 4 teeth with total sample size of (12 teeth divided into three groups)***.

Sample size was calculated again on G* power program version 3.1.7.9 to get the needed chart.

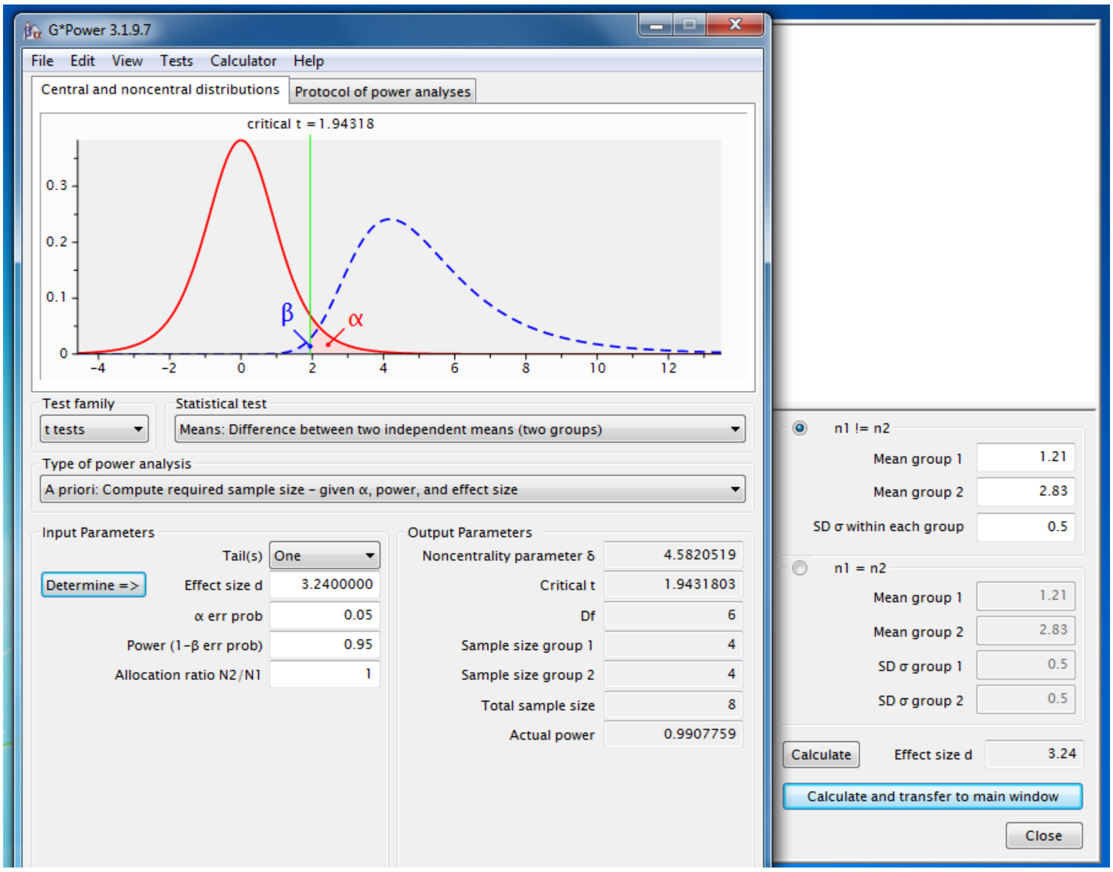

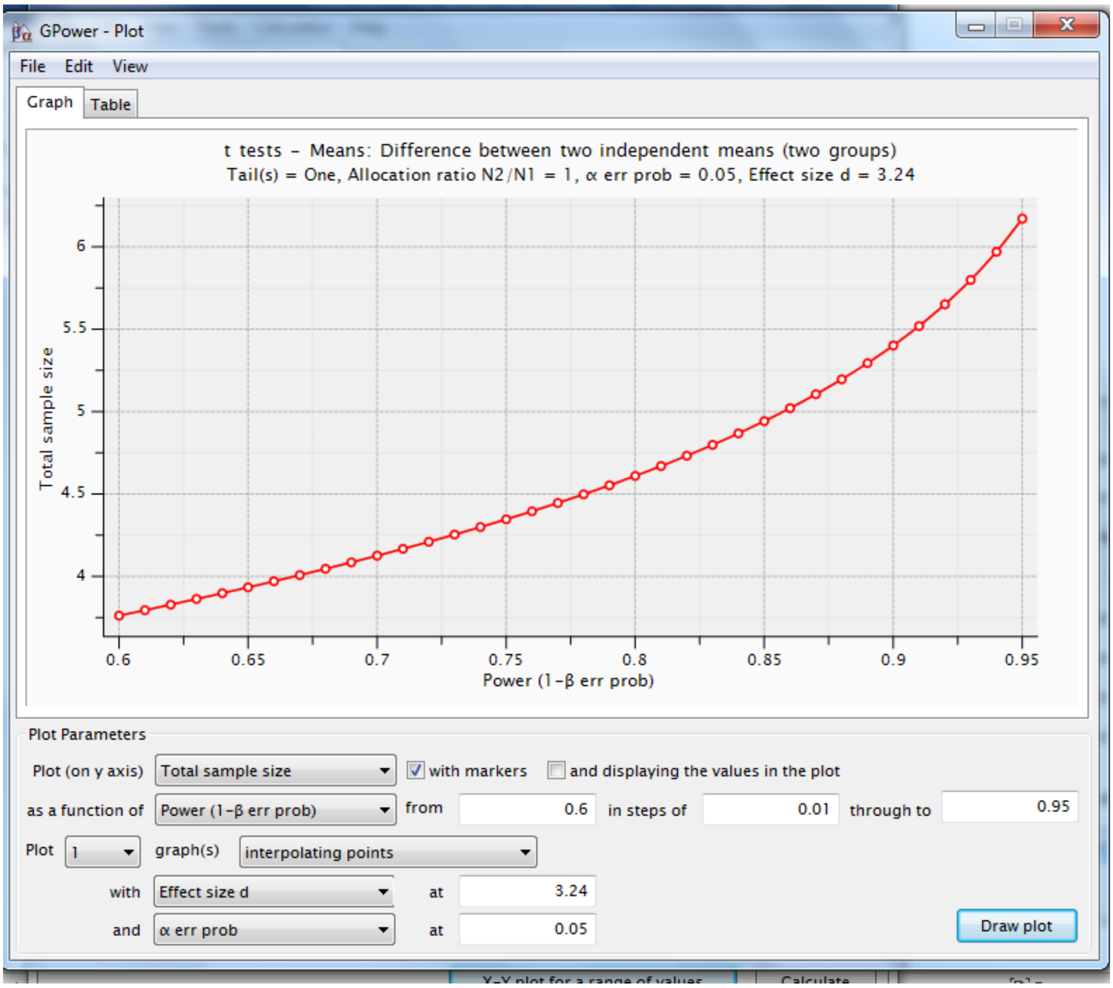
GPower - Plot
File Edit View
Graph
Table
t tests – Means: Difference between two independent means (two groups)
Tail(s) = One, Allocation ratio N2/N1 = 1, α err prob = 0.05, Effect size d = 3.24
Total sample size
6
5.5
5
4.5
4
0.6
0.65
0.7
0.75
0.8
0.85
0.9
0.95
Power (1–β err prob)
Plot Parameters
Plot (on y axis)
Total sample size
with markers
and displaying the values in the plot
as a function of
Power (1–β err prob)
from
0.6
in steps of
0.01
through to
0.95
Plot
1
graph(s)
interpolating points
with
Effect size d
at
3.24
and
α err prob
at
0.05
Draw plot
