# Supplementary figures and images for: Canal Transportation and Centring Ratio of Paediatric vs Regular Files in Primary Teeth
Source: Int Dent J. 2022 Oct 11;73(3):423–9. doi: 10.1016/j.identj.2022.09.003 (PMC10213759; doi:10.1016/j.identj.2022.09.003)

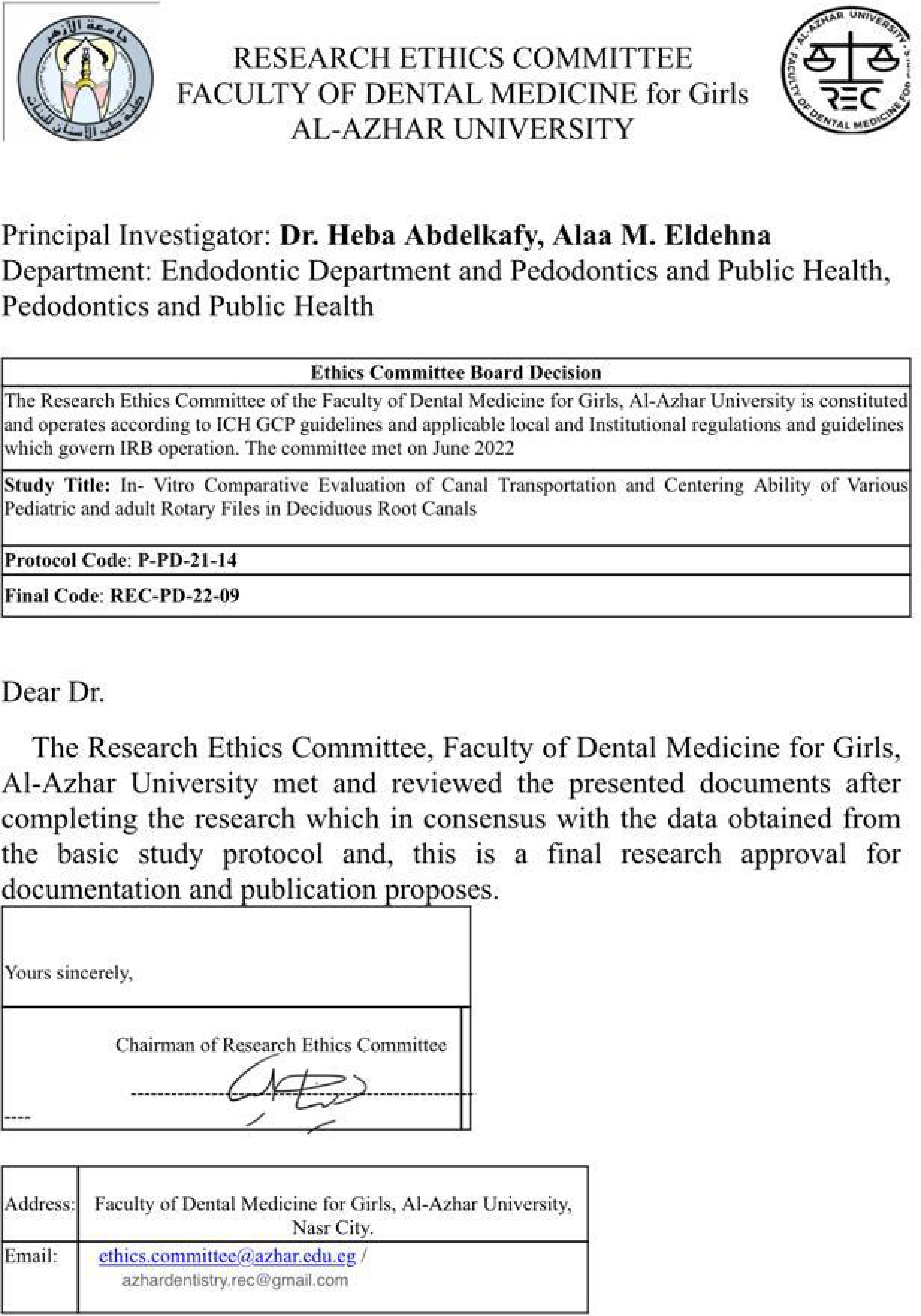

Supplement: Supplementary file 4 [file mmc4.jpg]
